# Exercise for the negative symptoms of schizophrenia: a review

**DOI:** 10.3389/fpsyt.2026.2001262

**Published:** 2026-10-09

**Authors:** Charles Trevelyan, Hina Mohsin

**Affiliations:** School of Medicine, California University of Science and Medicine, Colton, CA, United States

**Keywords:** adjunctive treatment, avolition, exercise, negative symptoms (schizophrenia), schizophrenia, yoga

## Abstract

Negative symptoms of schizophrenia, comprising avolition, asociality, anhedonia, blunted affect, and alogia, are a primary driver of functional impairment, tend to persist, and have no treatment approved specifically for them. Exercise has drawn interest as a low-cost, widely available, adjunctive intervention. This review synthesizes three recent evidence syntheses: an umbrella review of previously published meta-analyses and two network meta-analyses of randomized controlled trials. All three report significant reductions in negative symptoms, with yoga consistently the most effective modality; aerobic and mind-body exercise also show benefit, while the evidence for resistance exercise is inconsistent across analyses. Reported effect sizes range from small to large, and this divergence appears to reflect differences in comparator group and analytic method rather than differences in study quality. A dose-response analysis estimated an optimal total dose of 1200 metabolic equivalent minutes per week but proved unstable under sensitivity analysis. Certainty of evidence was rated low to very low throughout. Two constraints recur across the literature: the rating scales used cannot distinguish primary from secondary negative symptoms, and avolition itself likely limits sustained participation in exercise. Exercise is a reasonable adjunctive intervention, but its specific benefit for primary negative symptoms remains poorly understood.

## 1 Introduction

Schizophrenia is a severe mental disorder with a mean lifetime morbid risk of approximately 1%, causing a significant individual and societal burden (1). The prevalence, incidence, and disability-adjusted life years (DALYs) have all increased globally with 15.1 million DALYs in 2019, a 65% increase from 1990 (2). For individuals, unemployment is as high as 80-90% and life expectancy is reduced by 10–20 years (3). The main presentations of schizophrenia are generally divided into positive symptoms (hallucinations, delusions, thought disorganization) and negative symptoms (avolition, asociality, anhedonia, blunted affect, alogia). Positive symptoms may be intermittent and are responsive to antipsychotic medications. However, the negative symptoms are more likely to be chronic and have only a modest response to antipsychotics.

The negative symptoms are major drivers of functional impairment and poor quality of life (3). This review will cover the most recent evidence for the use of exercise as an intervention for treating negative symptoms and discuss challenges and gaps in the research.

## 2 Methods

This is a narrative review. PubMed was searched for articles published between 2000 and 2026 using combinations of the terms “schizophrenia,” “negative symptoms,” “exercise,” and “yoga,” limited to English-language publications. Priority was given to meta-analyses and systematic reviews of randomized controlled trials. Three evidence syntheses were selected for detailed comparison on the grounds that each was published between 2023 and 2025, each reported negative symptoms as a discrete outcome rather than as a component of total symptom scores, and together they represent three complementary analytic approaches: an umbrella review of previously published meta-analyses, a combined pairwise and network meta-analysis, and a Bayesian dose-response network meta-analysis. Additional sources were selected to provide background on the phenomenology, assessment, and pharmacological treatment of negative symptoms, and on proposed biological mechanisms. This review was not registered and was not conducted according to PRISMA guidance. No independent risk-of-bias appraisal was undertaken; certainty of evidence is reported as rated by the source reviews.

## 3 Characterizing negative symptoms

Negative symptoms have been described as an aspect of schizophrenia since at least 1919 (4) and have been thought to be pathophysiologically distinct from positive symptoms since at least 1974 (5). Negative symptoms are a distinct therapeutic indication and are separate from cognitive impairment. The main symptoms are accepted to be alogia, blunted affect, anhedonia, asociality, and avolition. According to DSM-5, diminished emotional expression and a decrease in self-initiated purposeful activities are the most common (6). Definitions are as follows (7):

Blunted affect: Decrease in the expression of emotion and reactivity to events as observed during the spontaneous or elicited expression of emotion (facial and vocal expression and expressive gestures).

Alogia: Reduction in quantity of words spoken and in spontaneous elaboration (i.e., amount of information spontaneously given beyond what is needed to answer a question).

Asociality: Reduced social interactions and initiative due to decreased motivation for and interest in forming and maintaining relationships with others.

Anhedonia: Reduced experience of pleasure for a variety of activities or events, during the activity or event and for future anticipated activities or events.

Avolition: Reduced initiation and persistence of goal-directed activity due to reduced motivation.

### 3.1 Primary and secondary symptoms

Importantly, negative symptoms can be categorized as primary or secondary. Primary symptoms have long been seen as a core part of schizophrenia, while secondary symptoms can be a result of positive symptoms, medication side effects, depression, or isolation. Secondary symptoms respond to treatment of the underlying cause, while primary ones are likely to persist even with antipsychotic treatment (8). In the case of primary and enduring negative symptoms, no treatments have shown robust efficacy (7).

### 3.2 Assessment of negative symptoms

Several different scales are used to evaluate negative symptom severity in the research context. The most widely used are the Scale for the Assessment of Negative Symptoms (SANS) and the Positive and Negative Syndrome Scale (PANSS) (9). Both include some aspects of cognition and disorganization in addition to negative symptoms and evaluate the avolition-apathy domain at the behavioral level only (7). In clinical practice, with careful evaluation over time, a more accurate characterization of the presence of primary versus secondary negative symptoms may be made.

### 3.3 Pharmacological treatments

Despite the lack of an FDA-approved treatment, many medications have been studied off-label for treating negative symptoms. These include antipsychotics, N-methyl-d-aspartate receptor (NMDAR) and other glutamatergic modulators, serotonin receptor (5-HTR) modulators, anti-inflammatory agents, antidepressants, pro-dopaminergic modulators (non-D2R antagonists), acetylcholine modulators, oxytocin, and phosphodiesterase (PDE) inhibitors (10). Of these, the most evidence-supported options are monotherapy with cariprazine, olanzapine, clozapine, and amisulpride, or adjunctive use of memantine, ondansetron, minocycline, and antidepressants. Muscarinic agonists such as xanomeline-trospium show promising preliminary results. Pooled across three RCTs in acutely exacerbated schizophrenia, xanomeline-trospium outperformed placebo on the PANSS negative subscale with a small effect (SMD -0.33, 95% CI -0.49 to -0.17), and a *post-hoc* analysis of a subgroup with prominent negative symptoms found a large effect, though in a small sample (n = 64; Cohen’s d = 1.18). The proposed mechanism is distinct from that of D2 antagonists: M1 and M4 agonism reduces presynaptic dopamine release in the associative striatum, while M1 stimulation raises cortical acetylcholine and secondarily dopamine, suggesting that patients with negative symptoms may benefit from increased regional cortical dopamine transmission (11). These results warrant caution, as all three trials enrolled acutely exacerbated patients and the negative symptom findings derive from *post-hoc* analyses, so improvement in secondary negative symptoms driven by positive symptom response cannot be excluded.

## 4 Overview of evidence for exercise

Given that current treatments exhibit a lack of robust efficacy, and there is no FDA-approved treatment for negative symptoms, there has been ongoing interest in exercise as a low-cost, widely available, and safe intervention. Since 2013, several meta-analyses have been conducted. These have typically included comparisons between studies investigating yoga, resistance exercise, aerobic exercise, resistance with aerobic exercise, and mind-body exercise including tai chi, Yijin Jing, and dance. There is high heterogeneity among studies that poses challenges for making comparisons.

A 2023 systematic review by Cella et al. considered 31 prior systematic reviews with meta-analyses of RCTs covering a range of psychosocial and behavioral interventions (12). Of the 31 meta-analyses, 6 addressed exercise therapies and included a total of more than 4,000 participants. Across these six reviews, reported effect sizes ranged from -0.59 to -0.24. One meta-analysis did not reach significance comparing yoga to exercise or treatment as usual (TAU), and two reported significance only when exercise was compared to TAU, but not to active controls. Five of the six found a significant reduction in negative symptoms with a largest effect size of –0.44. Three of the reviews considered mind-body exercises. In one, subgroup analysis found a small but significant effect favoring yoga, although this effect disappeared when studies at high risk of bias were excluded. Another concluded that the effects on negative symptoms were primarily driven by aerobic exercise (SMD= -0.31), though a separate review suggested that this result may likewise be affected by high risk of bias. Although many studies focused on positive symptom reduction, all included studies used a negative symptom scale, most often PANSS.

Cella et al. concluded that exercise therapy, as well as cognitive remediation, had the most robust evidence among all psychosocial and behavioral interventions, including CBT, showing a significant positive effect in reducing negative symptoms with a small-to-medium effect size. Among the samples included, mind-body exercises (including yoga) and aerobic exercise showed the largest effect sizes. Considering the causes for symptom reduction, the authors speculate that they may be complex, including not only the effects of physical exercise, but also the social aspects of the interventions.

In 2025, Wang et al. conducted a meta-analysis including 32 RCTs with 1,773 participants (13). They found that yoga, resistance exercise, aerobic exercise, and mind-body interventions all significantly reduced negative symptoms, with yoga and resistance exercise showing the largest effect. The effect sizes for yoga and resistance exercise were roughly two to three times those reported in earlier meta-analyses and umbrella reviews like Cella et al. Compared to no exercise control groups, the reported effect size for yoga was -1.14 and resistance exercise, -1.15.

Wang et al. employed both direct pairwise meta-analysis and network meta-analysis (NMA), which yielded different results. Direct pairwise meta-analysis found a significant improvement only with aerobic exercise (SMD=-0.87), mind body exercise (SMD: -0.94), and yoga (SMD: -0.92). However, NMA found all exercise modalities improved negative symptoms with resistance exercise (SMD: -1.15), yoga (SMD: -1.14), and aerobic exercise (SMD: -0.79) showing the largest effects.

### 4.1 Dose, duration, delivery

A 2024 meta-analysis by Yang et al. took the research a step further by investigating the dose-response relationship of exercise interventions to provide practical guidelines and recommendations for frequency and duration in clinical settings (14). This study focused on schizophrenia symptoms generally but included secondary outcomes for negative symptoms in 26 of 47 studies. Like Wang et al., they used NMA to indirectly compare modalities. They found significant improvement in negative symptoms with yoga (SMD: -1.01), resistance exercise plus aerobic exercise (SMD: -0.86), and aerobic exercise alone (SMD: -0.60). Yoga was also the most effective exercise in reducing total schizophrenia symptoms (SMD: -1.02).

They plotted the dose response relationship between overall dose and overall symptom reduction. The dose-response curve was modeled for total symptom change rather than for negative symptoms specifically. Improvements became significant at 340 metabolic equivalent minutes per week (METS-min/week), and the estimated optimal total exercise dose was 1200 METS-min/week (SMD: -0.956). On sensitivity analysis excluding high risk-of-bias studies, this fell to 690 METS-min/week and the effective range narrowed to 300-1000. Across the network meta-analysis as a whole, exercise not only reduced negative symptoms, but also improved depression indices, cognitive function, and quality of life.

**Table 1** summarizes the three reviews discussed above.

**Table 1:** Summary of the three evidence syntheses of exercise for negative symptoms of schizophrenia.

Review	Design	Studies on exercise/Participants	Effect size	Key caveat
Cella et al., 2023 (12)	Umbrella review of 31 studies	6 meta-analyses on exercise, n>4,000	Pooled exercise: -0.44 to -0.24	Included active controls, primarily addressed exercise collectively
Wang et al., 2025 (13)	Pairwise and NMA of 32 RCTs	32 RCTs, n=1,773	Yoga: -1.14 RE: -1.15 AE: -0.79	Larger effect size reflects NMA indirect comparisons, not risk of bias
Yang et al., 2024 (14)	NMA + dose response analysis of 47 studies	47 RCTs, n=4,031 (26 included negative symptoms, n=1276)	Yoga: -1.01 RE+AE: -0.86 AE: -0.60	Optimal dose of 1200 METS-min/week is for total symptoms

RE, Resistance Exercise; AE, Aerobic Exercise.

### 4.2 Proposed mechanisms

Considering pathophysiology, the strongest evidence supports dysfunction of dopaminergic neurotransmission as the cause of positive symptoms. This is the primary target of nearly all antipsychotic medications (3). However, it is thought that negative symptoms have a distinct etiology. More recent theories include an imbalance of inhibitory and excitatory neuronal transmission originating in the hippocampus (15). Other theories suggest dysfunction of serotonergic circuits, decreased neurogenesis, and inflammation (10).

Many of these factors have been shown to improve with exercise. Aerobic exercise increases neurogenesis, reduces inflammation, and improves hippocampal volume and function. It promotes increases in brain-derived neurotrophic factor and IGF-1, which can stimulate plasticity in the prefrontal cortex and hippocampus. Regular exercise also increases serotonin concentration in the brain, including in the hippocampus, which plays a role in learning, memory, and affect regulation. More research is needed, but these are possible explanations for the role exercise plays in reducing both negative and cognitive symptoms in schizophrenia (16).

For yoga, the additional benefit over other forms of exercise may be due to the maintained attention to breathing patterns and body position. These characteristics, as well as the social setting, may have effects similar to cognitive enhancement therapy, which has been shown to be beneficial to patients with schizophrenia (17).

## 5 Discussion

Taken together, these three reviews (12–14) converge on a small-to-medium positive effect of exercise on negative symptoms and begin to define an optimal dose range. NMA analysis of RCTs in Wang et al. and Yang et al. resulted in substantially larger effect sizes than those summarized by Cella et al. This divergence appears to be driven primarily by methodology and comparison group: network analysis allows for indirect comparisons, and Wang et al. used only no-intervention control groups. In contrast, several of the meta-analyses summarized by Cella et al. made comparisons against active controls which may have attenuated effect estimates. The indirect comparisons in NMA provide sufficient comparators where few studies have been conducted. Notably, resistance exercise showed a large significant effect in the Wang et al. network meta-analysis but did not reach significance in direct pairwise analysis or the Yang et al. NMA, underscoring how unstable the resistance-exercise effect remains and demonstrating the need for further research.

A risk-of-bias explanation for the increased effect sizes in the two NMAs is less well supported. In Wang et al., only 3 of 32 trials were rated at high risk of bias, and subgroup analysis by risk of bias did not produce significantly different outcomes (p = 0.076). In Yang et al., sensitivity analysis excluding high-risk-of-bias studies did not substantially change the effect estimates, although the authors note that minor changes did appear for total and negative symptoms.

The trials included in these reviews measured outcomes using total negative symptom scale scores, most often the PANSS negative subscale, SANS, or BNSS, none of which distinguish primary from secondary negative symptoms (9). Because exercise may improve depression, social isolation, and sleep, some portion of the measured benefit likely reflects improvement in secondary rather than primary negative symptoms (8). The speculation by Cella et al. that the social aspects of group-based interventions contribute to the observed effect is consistent with this possibility (12). Given that primary and enduring negative symptoms are precisely those for which no treatment has shown robust efficacy (7), trials that stratify participants by primary versus secondary status would substantially clarify where exercise is most useful. Cella et al. make the same observation, noting that negative symptom severity and the primary versus secondary distinction received limited attention across the reviews they surveyed (12).

Adherence and dropout were unevenly addressed. Only 9 of the 31 reviews surveyed by Cella et al. commented on attrition at all, and where reported it varied widely, reaching 32% in one exercise review (12). Wang et al. did record dropout in 30 of their 32 trials, 9 of which had attrition exceeding 20%, but they explicitly did not assess acceptability, tolerability, or safety, which they identified as a gap for future research (13). Given that exercise interventions depend on sustained participation, this limits conclusions about real-world feasibility in this population. Long-term follow-up data are similarly sparse, so the durability of any symptom improvement beyond the active intervention period remains unknown.

This concern is compounded by the nature of the target symptoms. Avolition poses a significant challenge to sustained participation in any exercise program. Yang et al. make a related point, noting that patients face multiple concrete barriers to exercise, including medication-related muscle pain and extrapyramidal symptoms, antipsychotic-induced fatigue and cardiovascular side effects, impaired coordination, obesity, and reduced motivation (14). Patients with the most severe negative symptoms may be the least likely to enroll, adhere, and complete an intervention, raising the possibility that trial samples are skewed towards patients with milder symptoms and that reported effect sizes overestimate what is achievable in practice. This has direct implications for real-world feasibility: supervised, structured, and socially embedded delivery models will be necessary to make exercise viable for the patients who stand to benefit most.

Concerning exercise selection, frequency, and duration, more research is needed to make specific clinical recommendations. The Wang et al. NMA found no significant difference between exercise modalities, only between modalities and controls, implying that, at this time, individual preference may be a primary guide in treatment planning. Yoga may be of particular benefit to patients already stable on antipsychotic medication (18, 19). To further this point, subgroup analysis found that age had a significant impact on outcome, with the strongest results in patients aged 31–40 years. This could be due to younger individuals having a greater tolerance for exercise as well as being earlier in the disease course. Extending this logic, exercise has been proposed as a protective factor for individuals at clinical high risk for psychosis, including first-degree relatives and those showing early prodromal signs (7, 18, 20). Effect size also varied by location, again suggesting that exercises tailored to the regional demographic, and to the individual, may produce the best results (13). It should be emphasized that the dose recommendation is a modeled estimate rather than an established clinical recommendation. It has not been prospectively validated, and no professional body currently endorses a specific exercise dose for negative symptoms specifically.

## 6 Limitations

All the meta-analyses cited challenges arising from high heterogeneity of studies. There was some inclusion of RCTs that were at high risk of bias. Bias due to a lack of blinding was not assessed. Effects at follow-up were not included due to high variability between studies. Formal certainty ratings were correspondingly low: Wang et al. graded most direct and indirect comparisons as low certainty under GRADE, and Yang et al. rated 97.2% of the evidence for their primary outcome as very low certainty under CINeMA.

There were very few North American studies included in the analyses (only 2 of 32 studies included in the Wang et al. meta-analysis), which limits generalizability due to cultural aspects.

## 7 Conclusion

Exercise has been shown to have a modest positive effect on the negative symptoms of schizophrenia. Given the well-established positive effects of exercise on many health metrics, and despite consideration of the heterogeneity of current research, it is reasonable to consider exercise as an adjunctive intervention targeting negative symptoms in clinical practice, pending more rigorous, low-risk-of-bias trials. Given that the exercise modalities discussed have all shown some degree of efficacy, treatment could be tailored to what best suits the individual patient, which may increase adherence despite the challenges posed by the negative symptoms themselves. Further research is needed to improve generalizability, investigate safety and tolerability, and address the effectiveness, feasibility, and accessibility of exercise treatment for severe negative symptoms.
